# Prediction of emergency department revisits among child and youth mental health outpatients using deep learning techniques

**DOI:** 10.1186/s12911-024-02450-1

**Published:** 2024-02-08

**Authors:** Simran Saggu, Hirad Daneshvar, Reza Samavi, Paulo Pires, Roberto B. Sassi, Thomas E. Doyle, Judy Zhao, Ahmad Mauluddin, Laura Duncan

**Affiliations:** 1 Department of Health Research Methodology, Evidence & Impact, McMaster University, 1280 Main St W, Hamilton, Ontario L8S 4K1 Canada; 2 Department of Electrical, Computer and Biomedical Engineering, Toronto Metropolitan University, 350 Victoria Street, Toronto, Ontario M5B 2K3 Canada; 3 Department of Psychiatry & Behavioural Neurosciences, McMaster University, 1280 Main St W, Hamilton, Ontario L8S 4K1 Canada; 4 McMaster Children’s Hospital, Hamilton Health Sciences, 1200 Main St West, Hamilton, Ontario L8N 3Z5 Canada; 5 Department of Psychiatry, University of British Columbia, UBC Vancouver Campus, Vancouver, BC V6T 2A1 Canada; 6 Department of Electrical & Computer Engineering, McMaster University, 1280 Main St W, Hamilton, Ontario L8S 4K1 Canada

**Keywords:** Mental health, Machine learning, Graph neural network, Deep learning, Emergency department, Revisits, Prediction

## Abstract

**Background:**

The proportion of Canadian youth seeking mental health support from an emergency department (ED) has risen in recent years. As EDs typically address urgent mental health crises, revisiting an ED may represent unmet mental health needs. Accurate ED revisit prediction could aid early intervention and ensure efficient healthcare resource allocation. We examine the potential increased accuracy and performance of graph neural network (GNN) machine learning models compared to recurrent neural network (RNN), and baseline conventional machine learning and regression models for predicting ED revisit in electronic health record (EHR) data.

**Methods:**

This study used EHR data for children and youth aged 4–17 seeking services at McMaster Children’s Hospital’s Child and Youth Mental Health Program outpatient service to develop and evaluate GNN and RNN models to predict whether a child/youth with an ED visit had an ED revisit within 30 days. GNN and RNN models were developed and compared against conventional baseline models. Model performance for GNN, RNN, XGBoost, decision tree and logistic regression models was evaluated using F1 scores.

**Results:**

The GNN model outperformed the RNN model by an F1-score increase of 0.0511 and the best performing conventional machine learning model by an F1-score increase of 0.0470. Precision, recall, receiver operating characteristic (ROC) curves, and positive and negative predictive values showed that the GNN model performed the best, and the RNN model performed similarly to the XGBoost model. Performance increases were most noticeable for recall and negative predictive value than for precision and positive predictive value.

**Conclusions:**

This study demonstrates the improved accuracy and potential utility of GNN models in predicting ED revisits among children and youth, although model performance may not be sufficient for clinical implementation. Given the improvements in recall and negative predictive value, GNN models should be further explored to develop algorithms that can inform clinical decision-making in ways that facilitate targeted interventions, optimize resource allocation, and improve outcomes for children and youth.

**Supplementary Information:**

The online version contains supplementary material available at 10.1186/s12911-024-02450-1.

## Background

Child and youth (herein child/youth) mental health disorders affect approximately 1 in 5 Canadian children/youth and left untreated can lead to chronic difficulties and negative downstream effects [1, 2]. In Canada, increased demand for mental health services includes children/youth seeking immediate support for mental health-related concerns through an emergency department (ED) [3, 4]. The total number of pediatric ED visits declined after the onset of the COVID-19 pandemic, but the proportion of all visits that were for mental health increased [5].

Revisiting an ED, typically within a 30-day window, is a commonly used healthcare utilization metric used to assess the effectiveness of ED-based interventions [6]. Research on pediatric mental health-related ED revisits in BC reported decreases in high-acuity visits and increases in mid-acuity level visits [7]. Mental health support in an ED provides immediate safety for emergent conditions through crisis intervention or referrals to specialized or community resources. Individuals with lower acuity may opt to visit an ED because appropriate community resources were unavailable in the needed timeframe. Visiting an ED when less resource-intensive services may be clinically appropriate could lead to wasted or misdirected resources [8]. Accurate prediction of children/youth who are at risk of an ED revisit could allow for early identification and targeted interventions to prevent the escalation of crises and increased acuity. From an organizational perspective, better targeting of the response in accordance with acuity level increases efficiency in the allocation of healthcare resources.

Machine-based algorithmic approaches show promise for tapping into the predictive value of electronic health record (EHR) data to support child/youth mental health [9, 10]. In contrast with traditional machine learning, or ‘shallow’ learning, deep learning uses artificial neural networks—inspired by the organization and function of the brain—to extract meaningful patterns and representations from complex data. Recurrent neural network (RNN) models process sequential and time series medical history and visit data over time, focusing on the ordering of events but not accounting for bidirectional relationships. RNN models consider past inputs in predictions that accurately capture temporal dependencies in the data [11]. Graph neural network (GNN) approaches go further in accounting for temporal, interdependent, and multidirectional relationships in EHR data by using graph structures to capture complex relationships between symptoms, treatments, and patient characteristics. Graphs are mathematical representations of networks composed of nodes (different variables or features representing individuals, objects, or concepts) and edges (connections or relationships between variables illustrating how nodes are linked within the graph) [12]. While RNN models capture event order, GNN models consider event order, other types of connections (e.g. spatial relationships, multimodal dependencies, semantic associations), and the influence these connections have on each other. Despite offering a better fit to EHR data, the application of GNN models to ED revisit prediction is underexplored.

Predictive modeling for revisit, readmission and other health encounter prediction has used traditional logistic regression models, classical machine learning models and, to a lesser extent, deep learning models including RNNs and GNNs. Regression has been used to predict 72-hour, 9- and 30-day revisits with model performance ranging from AUROC = 0.741(area under the receiver operating characteristic curve (AUROC) values range from 0 to 1 and indicate the model’s overall correctness) to C-statistic (analogous to AUROC) = 0.773 [13–16]. Classical and ensemble models applied to health encounters include tree-based models, voting classifier models, neural networks, regularized logistic regression, gradient boosting and support vector machines [17–19]. A scoping review of traditional machine learning methods predicting readmission reported a median AUROC of 0.68 [20].

Deep leaning techniques include the application of RNN models to predict ICU readmission with AUROC = 0.74 to 0.79 [21, 22] and hospital readmission among congestive heart failure patients (AUROC = 0.77) [23], diabetic patients (AUROC = 0.80) [24], and lupus patients (AUROC = 0.66) [20, 25]. These models outperform comparable machine learning and baseline regression models within their respective studies. While not used for ED revisits specifically, GNN models show promise for other types of health encounter prediction [26]. A multimodal spatiotemporal GNN model was developed to predict all-cause hospital readmission, outperforming the clinical reference standard and other baseline models (AUROC: 0.79) [27], and a DeepNote-GNN model outperformed baseline models with an AUROC of 0.80 for 30-day hospital readmission prediction [28].

In the field of mental health, a GNN model was applied to mobile sensing data for the early detection of anxiety and mood disorders, achieving improvements of 0.067 in AUROC compared to the best performing baseline model [29].

In the prediction of child/youth ED revisits, GNN models are an emerging approach with the potential to improve prediction accuracy and enhance the utility of models—hence the focus of our study. Our goal was to determine whether GNN models can be applied to child/youth EHR data to predict 30-day ED revisit from a sample of children/youth who were in contact with mental health outpatient services between 2011 and 2021 and who had at least one ED visit between 2002 and July 2021. Our objective was to develop a GNN model to predict 30-day ED revisit and compare model performance against RNN, conventional machine learning and logistic regression models.

By developing and evaluating a GNN model for predicting ED visits among children/youth, this study uses a new approach that could contribute to improving child/youth outcomes and optimizing healthcare resource allocation. Our results can inform the development of AI-assisted decision-making tools for healthcare providers, enabling early identification of at-risk children/youth and targeted interventions to reduce ED visit and revisit rates. The application of these advanced computational approaches can contribute to a better understanding of the complex dynamics involved in child/youth health service utilization, ultimately informing policy and clinical decision-making processes.

## Methods

### Dataset

**Fig. 1:**
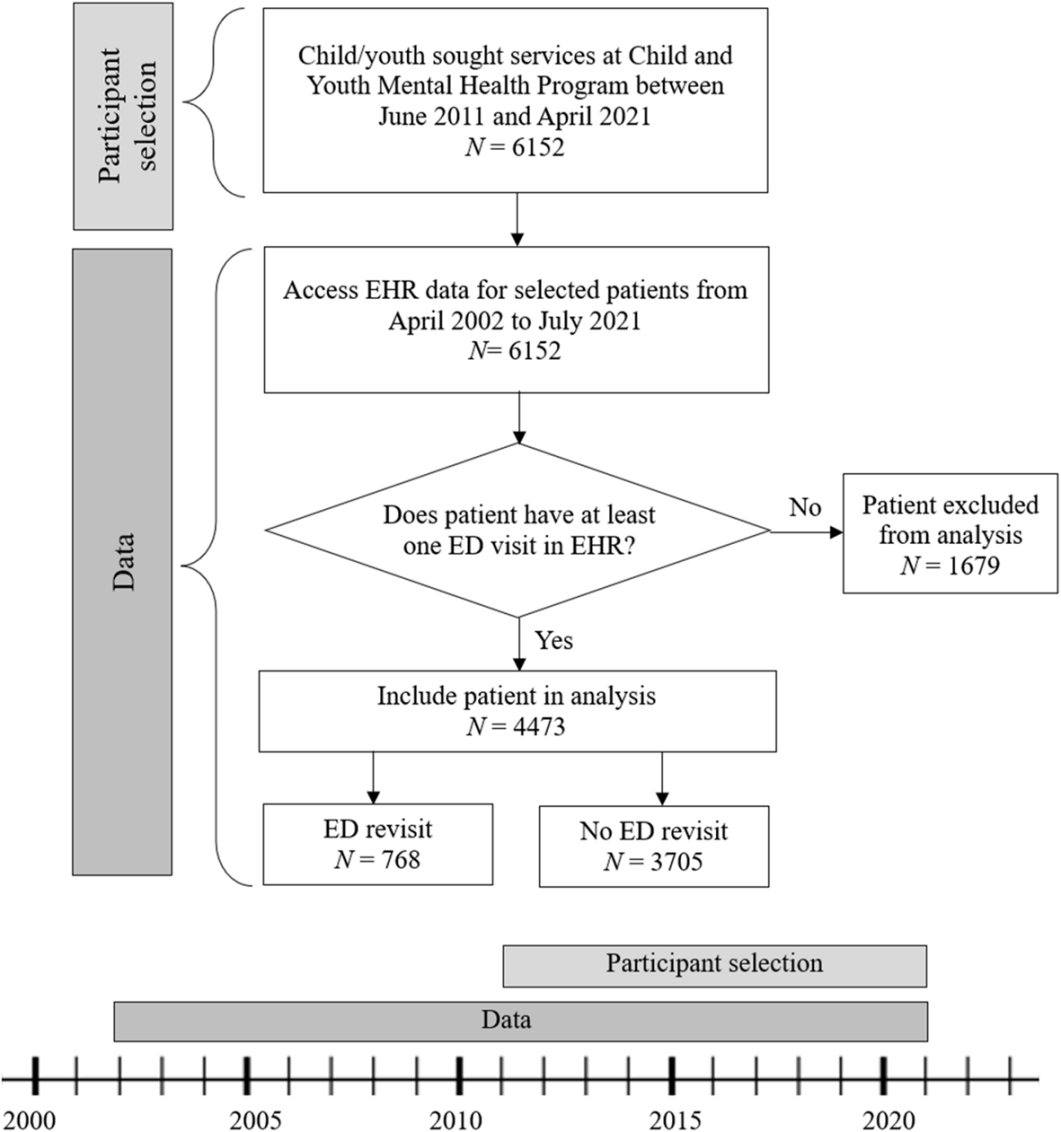
Participant selection, data and timeline

McMaster Children’s Hospital (MCH) is one of Canada’s largest pediatric hospitals serving South-Central Ontario offering inpatient, outpatient and ED mental health care to children and youth. While most children/youth dealing with mental health difficulties are discharged from the ED with referrals to outpatient services, some are admitted for inpatient care before being referred for follow-up care. While receiving outpatient services, children/youth may visit or revisit the ED for urgent care. The study used retrospective administrative health records for 6152 child/youth outpatients aged 4 to 17 who sought services at MCH’s Child and Youth Mental Health Program outpatient service between June 2011 and April 2021. These child/youth’s medical record numbers were linked to their administrative health records from April 2002 to July 2021 in the Meditech EHR system, including inpatient, outpatient, and emergency department visit data for MCH-based services. From these 6152 children/youth, the National Ambulatory Care Reporting System (NACRS) data were used to identify those who had at least one ED visit (*N* = 4473). Of these, 768 revisited the ED within 30-days of a previous visit. Figure 1 shows the participant selection process, dataset and timeline. All data used in the study were deidentified and access was approved by the Hamilton integrated Research Ethics Board (HiREB no. 8114). Procedures are reported according to the International Journal of Medical Informatics checklist for assessment of medical AI [30].

### Prediction target

Our prediction target was a binary indicator of 30-day ED revisit (coded as revisited/not revisited). Each visit had an associated indicator column for whether a child/youth had an ED revisit within the next 30 days. While restricting ED visits and revisits to mental health-related diagnoses was considered, we included any ED visits and revisits for the following reasons: 1) ICD-10 codes (used for most responsible diagnosis) underrepresent child/youth psychiatric disorders [31]; 2) diagnostic codes for mental health and substance use disorders used in administrative health records are unreliable [32, 33]; 3) most responsible diagnosis and presenting complaint misses any secondary mental health-related diagnoses that might be a contributing reason for the visit. Instead, we use EHR data from mental health outpatients expecting that they may be at increased risk for visiting an ED for mental health-related concerns compared to children/youth who did not receive outpatient services during the study timeframe.

### Features

Child/youth patient visits were listed in date order. Each visit was accompanied by the feature descriptions shown in Table 1 including: 1) child/youth’s age in years at time of visit; 2) triage level coded as a categorical variable based on the type and severity of initial presenting signs and symptoms using the Canadian Triage Acuity Scale (CTAS) ranging from 1 (Resuscitation) to 5 (Non-urgent), with 9 indicating an unknown triage level; 3) visit disposition identifying the type of separation from the ambulatory care service after registration including 20 possible categories of types of transfer (10 sub-categories), going home (2 sub-categories), leaving the hospital (4 sub-categories), and death (4 sub-categories); 4) most responsible diagnosis capturing the most clinically significant reason for the child/youth’s ED visit based on the ICD-10 coding system and including 1377 categories; and 5) the 61 types of service(s) of the health professional(s) responsible for the child/youth during the visit. All features were static and attached to each visit. Date of visit was used to temporally order visits and the order (not date or time between visits) was captured in the patient graph. One drawback of GNN models is that due to their complexity and ability to capture non-linearity in the data, they are inherently non-interpretable. Using interpretability techniques to extract feature importance metrics was beyond the scope of our study.

**Table 1:** Feature descriptions

Feature	Type	Attributes
Child/youth's age in years at time of visit	Numeric	Mean = 12.4 s.d = 3.9 Min = 4 Max = 18
Triage level	Categorical	5 categories
Visit disposition code	Categorical	20 categories
Most responsible diagnosis code	Categorical	1377 categories
Type of service code	Categorical	61 categories

### Pre-processing

The dataset was cleaned by the hospital Decision Support department who is responsible for the quality and integrity of EHR data. Data pre-processing involved generating an indictor column for 30-day revisit categorized as ‘1’ if the last visit was within the next 30 days (revisited), and ‘0’ otherwise (not revisited). For children/youths with only one visit to the ED, the indicator column was set to ‘0’. For children/youth with more than one visit, the last visit was removed from the input data and the second last visit (*n*-1) was used to create a binary outcome variable for the data to be trained on. The number of visits per child/youth was also limited to 10 (this number was selected as the average number of visits was 5 with a standard deviation of 5), so only the last 10 visits (before visit *n*) were kept as input data. Selected features had no missing data. Feature pre-processing included one-hot encoding of categorical features and the normalization of age.

### Model development

**Fig. 2:**
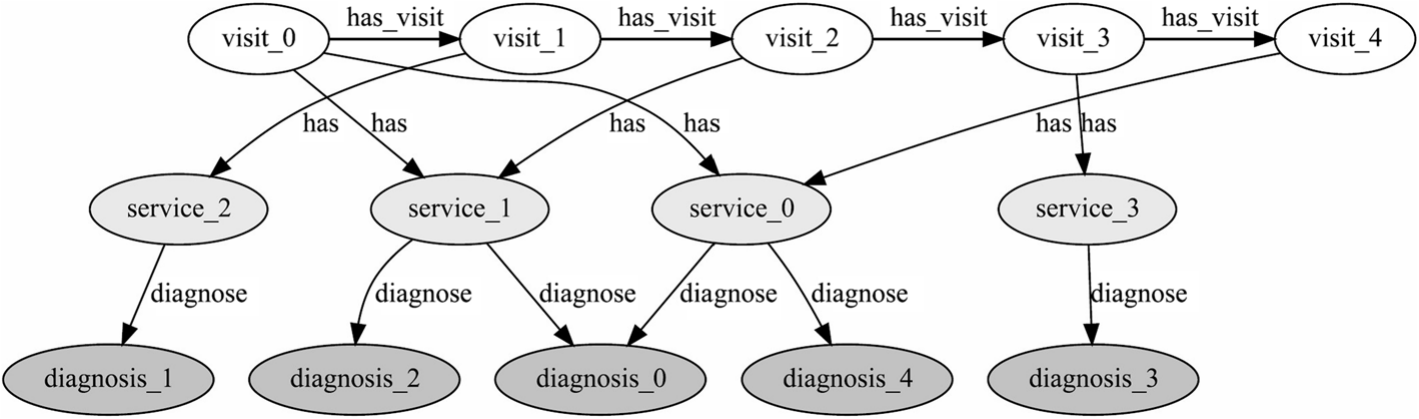
An example of a graph for a child/youth with five visits

The GNN and RNN models were identified by Daneshvar et al., [34] from a set of candidate models as the best performing. Models were developed using supervised learning, were trained with similar parameters, and were constrained to one layer to ensure model comparability. For the GNN model, a graph was constructed for each child/youth using the identified features—Fig. 2 shows an example graph. Different types of connections were established between nodes, such as visit-visit, visit-service, and service-diagnosis connections. Each patient graph had an adjacency matrix and a node feature matrix which was fed to the GNN. The GNN allowed aggregate node representations in a single graph representation and used this representation for classifications. The graphs accommodate multiple visits, getting larger or smaller depending on the visit number. While the structure of the graph is fixed, the number of nodes depends on the number of visits.

For the RNN model, the sequency of visit information was fed into the model with multi-hot encoding used to handle multiple visit features. For the conventional machine learning and logistic regression models, all visits were concatenated into a single row and retained temporal ordering. Detailed technical specifications of GNN and RNN models, operators, calibration and software used can be found in the Appendix and in Daneshvar et al. [34] XGBoost, decision tree and logistic regression models were selected as baseline comparison models.

### Data balancing

The dataset was highly imbalanced—the group with an ED revisit was significantly smaller (*N* = 768) than the group without an ED revisit (*N* = 3706)—which can cause classification inaccuracy with a bias toward the majority group. To address this, undersampling techniques were used to create five data subsets and the model was trained and evaluated using five-fold cross-validation for each subset following best practice [35]. First, five random samples of 768 unique children/youth from the group without an ED revisit were selected. Each random sample was separately merged with the 768 children/youth with an ED revisit to create a separate balanced dataset, resulting in five balanced datasets. For each of these a five-fold cross validation approach was used to train and evaluate the models, in which the dataset was split into five equally sized ‘folds’. In each step of the five-fold cross validation, four of the five folds are selected as the training set and the remaining fold as the test set. This process is repeated until each fold has been used as the test set exactly once. A performance score was obtained for each fold. A single performance metric representing the model’s overall performance was generated by averaging the 25 scores.

### Model training

GNN and RNN models were trained on the data for a total of 300 epochs (a complete iteration through a dataset) in batches of 500 (the number of data points that are simultaneously processed by the training algorithm during each epoch). Optimization algorithms were used to fine-tune the models and improve performance.

### Model evaluation

Performance was assessed using F1 scores which are expressed as a value between 0 and 1, where a higher score indicates better model performance and scores of 0.7 and higher are commonly used as a threshold for ‘good’ model performance [36]. F1 scores consider trade-offs between precision and recall and are better able to differentiate between different types of errors made by the model than AUROC [11]. Precision represents the proportion of correctly predicted positive cases (true positives) out of all *predicted* positive cases (true positives + false positives), while recall represents the proportion of true positives out of all *actual* positive cases (true positives + false negatives). For interpretability, AUROC, % accuracy, and positive and negative predictive values (PPV, NPV) were also generated.

## Results

Of the 6152 children/youth in our dataset, 73% had at least one ED visit (*N* = 4473). Of these, 768 (17%) revisited the ED within 30 days. The average age of children/youth with at least one ED visit was 12.34 years (SD = 3.9) and the median age was 14 years. Gender and other socio-demographic characteristics were unavailable in the administrative health data. Based on summaries of outpatient intake assessments completed by caregivers and youth, most children are born in Canada (~ 97%), speak English at home (~ 98%), and approximately 25% identify as Black, Indigenous or a person of colour. Approximately 80% of caregivers reported being informed that their child/youth had a mental health disorder and approximately 50% reported that their child/youth was currently using prescribed medications for mental health concerns.

**Fig. 3:**
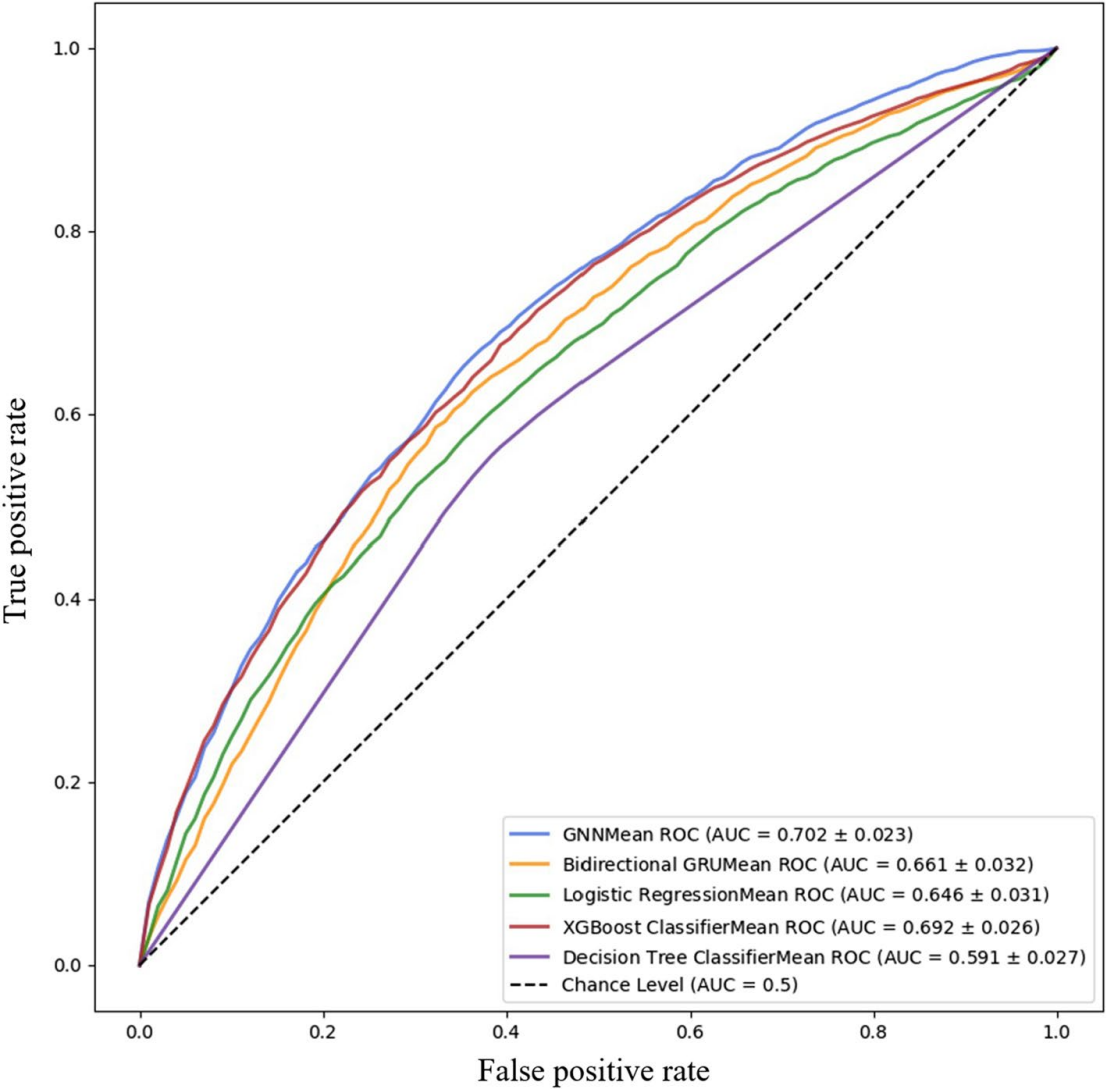
Receiver operating characteristic (ROC) curves for the GNN, RNN, conventional machine learning and logistic regression classification models

Model performance metrics are shown in Table 2. For the prediction of a 30-day ED revisit within 30 days for children/youth with any ED visit, F1 scores for the GNN model and RNN model were .6502 and .5991 respectively. XGBoost and decision tree model F1-scores were .6032 and .5779 respectively, and the baseline logistic regression model’s F1-score was .5828. The XGBoost model had similar F1 score and accuracy to the RNN model and had better recall, AUROC, PPV and NPV, but not better precision. The GNN model outperformed the XGBoost model on all metrics except precision and PPV. ROC curves were generated and are shown in Fig. 3. The difference between GNN and RNN model F1 scores, recall, AUROC and accuracy were statistically significant based on *t*-tests at *p* < .05. Precision was not statistically significantly different.

**Table 2:** Performance metrics of GNN, RNN, conventional machine learningand logistic regression classification models

Model type	F1 scores (SD)	Precision	Recall	AUROC	Accuracy (%)	PPV (%)	NPV (%)
GNN	.6502 (.0356)	.6369	.6654	.7022	64.43	64.24	65.37
RNN	.5991 (.0430)	.6502	.5566	.6611	62.82	64.76	61.03
XGBoost	.6032 (.0437)	.6414	.5707	.6919	62.89	65.49	62.08
Decision tree	.5779 (.0462)	.5838	.5736	.5908	58.64	59.36	58.76
Logistic regression	.5828 (.0415)	.6166	.5538	.6455	60.58	62.22	59.84

*AUROC* Area under the Receiver Operating Characteristic curve, *SD* standard deviation, *PPV* positive predictive value, *NPV* negative predictive value

## Discussion

Evidence points to the potential benefits of developing predictive tools to support diagnostic, prognostic and treatment decisions in child/youth mental health [10]. Deep learning RNN models can consider the sequence of visits but GNN models also consider interconnections between different types of information (i.e. features). This provides opportunities for developing prediction models that better represent the structure of administrative health data and so may better inform the development of models that help to target interventions aimed at reducing revisit rates, potentially improving healthcare resource planning and allocation in child/youth mental health.

This study demonstrates the increased ability of GNN models to capture complexities in EHR data and their potential value and improved performance for predicting ED revisits among children/youth compared to RNNs, baseline conventional machine learning and regression models. The GNN model outperformed the RNN model by .0511 in F1 scores—a moderate improvement. The XGBoost model performance was similar to the RNN model. Model accuracy did not reach the commonly used 0.7 threshold meaning that the performance may not be sufficient for clinical implementation without further model refinement. Other studies of treatment outcomes in child/youth mental health are scarce but those that exist report accuracies of over 0.8 [37, 38]. RNN models for hospital and ICU readmission prediction report AUROC values ranging from 0.66 to 0.80 [23–25]. Our best performing models had an AUROC of 0.70 which is in line with existing models.

Precision and positive predictive value of GNN, RNN and XGBoost models were comparable, but the performance improvement of the GNN model was more evident for recall and negative predictive value. While the models predict which children/youth will have an ED revisit with similar performance, the GNN model was best able to predict which children/youth will *not* revisit the ED. This is important when considering the clinical implications of incorrectly identifying a child/youth at risk for revisit and providing some intervention or treatment that may not be needed.

The strengths of this study include the following. First, our study has a large sample size in a field that typically suffers from small samples, despite focusing on a single site and a rare event (i.e. ED revisit). Second, external validation of the model will be possible in nationally available NACRS data. Externally validating these models independently with new individuals will reveal the extent to which models are generalizable to other settings, whether model performance improves in provincial or national data, and whether wider application is feasible. Third, we apply novel deep learning models in a field where these types of models have not been used, generating new evidence about their potential utility. The findings suggest that GNN models leverage the relationships present in patient EHR data leading to improved model performance compared to baseline models.

There are also study limitations. First, due to the lack of valid and reliable case definitions for mental health-related ED visits in EHR data, it was not possible to develop models specific to mental health-related reasons. This is a challenge faced by all prediction models that rely only on diagnostic codes in EHR data. While it will be important to determine whether GNN models can be developed for mental health-related ED revisits, this was beyond the scope of our current project. Another area for future exploration is the extension of deep learning models to predict any ED visit (not only revisit) among children/youth who both do and do not visit the ED. This requires data fusion to combine ED visit data with other EHR and non-EHR-data that are collected for all patients, not only those accessing the ED. Data fusion comes with its own challenges [39] but being able to identify children/youth more likely to visit the ED has clinical utility. Data fusion would also allow access to demographic characteristics other than age, which were not available in the EHR data we used and so limited exploration of their influence on ED utilization. Second, the lack of GNN and RNN models in child/youth ED revisit prediction means we are limited in our ability to evaluate model performance against other models. It is unknown if the threshold of F1 > 0.7 is a reasonable one in this context. A lower criterion may be appropriate and indicative of clinical utility. Third, our data includes visits that occurred during the COVID-19 pandemic when ED visit patterns changed [40]. However, only 2 of the 19 years of data accessed were during the pandemic. Further validation of the model in post-pandemic data will be important, although it is likely that healthcare pressures resulting in different visit patterns and demands still exist even though the acute stage of the pandemic has passed. Finally, given the complexity level of GNN models, it was not possible to export feature importance information which can provide insights into the most important features in the prediction, particularly when comparing models. The benefits GNNs offer in their ability to better capture complex, non-linear, visit data comes at the cost of easy interpretability. Interpretability techniques are being developed for deep learning models and finding appropriate approaches is an important area for further investigation. Future work needed across all applications of machine learning relate to: 1) model fairness and bias; 2) acceptability and ethics of using machine learning models with data about children, youth and their families; and 3) education and training of clinicians to ensure potential model users understand how predictive models have been developed and their potential limitations.

## Conclusions

This is an exciting time for the exploration of machine learning prediction models to enhance clinical decision-making. This study demonstrates the improved accuracy and potential utility of GNN models in predicting ED revisits among children and youth. Given the improvements in recall and negative predictive value, GNN models should be further explored to develop algorithms that can inform clinical decision-making in ways that facilitate targeted interventions, optimize resource allocation, and improve outcomes for children and youth.

### Supplementary Information

**Additional file 1.**

## Data Availability

There are ethical restrictions on sharing the study data as the data contains potentially sensitive information. It is not possible to share the data due to these ethical restrictions. Model code is available from the authors upon request.

## Abbreviations

AUROC: Area under the Receiver Operating Characteristic curve
BC: British Columbia
CTAS: Canadian Triage Acuity Scale
ED: Emergency department
EHR: Electronic health record
GNN: Graph neural network
HiREB: Hamilton integrated Research Ethics Board
MCH: McMaster Children’s Hospital
NACRS: National Ambulatory Care Reporting System
NPV: Negative predictive value
PPV: Positive predictive value
RNN: Recurrent neural network
SD: Standard deviation
