# Supplementary material for: Prediction of emergency department revisits among child and youth mental health outpatients using deep learning techniques
Source: BMC Med Inform Decis Mak. 2024 Feb 8;24:42. doi: 10.1186/s12911-024-02450-1 (PMC10854017; doi:10.1186/s12911-024-02450-1)
Supplement: Supplementary file 1 — Additional file 1. [file 12911_2024_2450_MOESM1_ESM.docx]

**Appendix**

**Technical information**

- Python version 3.9 and PyTorch version 1.10.0
- GPU (NVIDIA GeForce GTX 1650 Ti with CUDA version 11.4
- The optimization of the networks was performed using the Adam Optimizer with an ExponentialLR Scheduler.
- Heterogenous (bipartite) patient graphs were used which, compared to homogenous graphs that only consider a single type of data, can better capture complex healthcare dynamics by considering interconnections among multiple factors.

**RNN and GNN model development**

The model type, description and source are shown in Table A1. Candidate GNN models were identified based on two criteria: 1) models were bipartite (a mathematical feature where nodes and edges are connected allowing for increased model complexity), and 2) the recency of the operator development (preference given to more recently developed, and innovative, operator)

Table A1. Candidate model types

| Model type | Model | Description | Source |
| --- | --- | --- | --- |
| GNN | PTO | Proposed GNN architecture with a Transformer based graph | <https://arxiv.org/abs/2009.03509> |
| RNN | Bi-GRU | Bidirectional Gated Recurrent Unit | https://arxiv.org/abs/1406.1078 |

**Model calibration**

Reliability diagrams are a visual approximation of a models’ confidence calibration. Confidence calibration plots the predictive probabilities (i.e., confidence) against its predictive accuracy. If a model correctly predicts classifications with a confidence of 0.67 and is well calibrated, it would also have 0.67 accuracy. Reliability diagrams chart mean confidence on the x-axis and accuracy within each bin on the y-axis for equally spaced bins. For each bin, bar height corresponds to the calculated mean accuracy of the predictions within that bin, showing how well the predicted probabilities align with the actual outcomes. A well-calibrated model would have bars that align well with the diagonal line ($y=x$).

**Expected Calibration Error (ECE)**

Expected Calibration Error (ECE) is a scalar measure that summarizes the difference between the model’s confidence and accuracy.^1^ ECE is weighted average of the difference between the accuracy and confidence of each bin. Models with lower ECE values are more reliable. The reliability diagram and the ECE values of the best performing GNN, RNN, classical machine learning and logistic regression classifiers for ten bins are shown in Figure A1. The blue bars represent the accuracy within each bin, and the red bars represent the gap (difference) between the classifier’s prediction accuracy and the average prediction confidence within each bin, i.e., the calibration gap. The XGBoost model has the lowest ECE value of 0.1758, followed by the GNN model at 0.1810, followed by the decision tree model, logistic regression and RNN model. In general, deep learning models tend to be overconfident but GNNs specifically have been shown to be underconfident and needing calibration.^2,3^ Questions about model trustworthiness are beyond the scope of the current study. Future work will compare calibration techniques to improve model confidence.

**
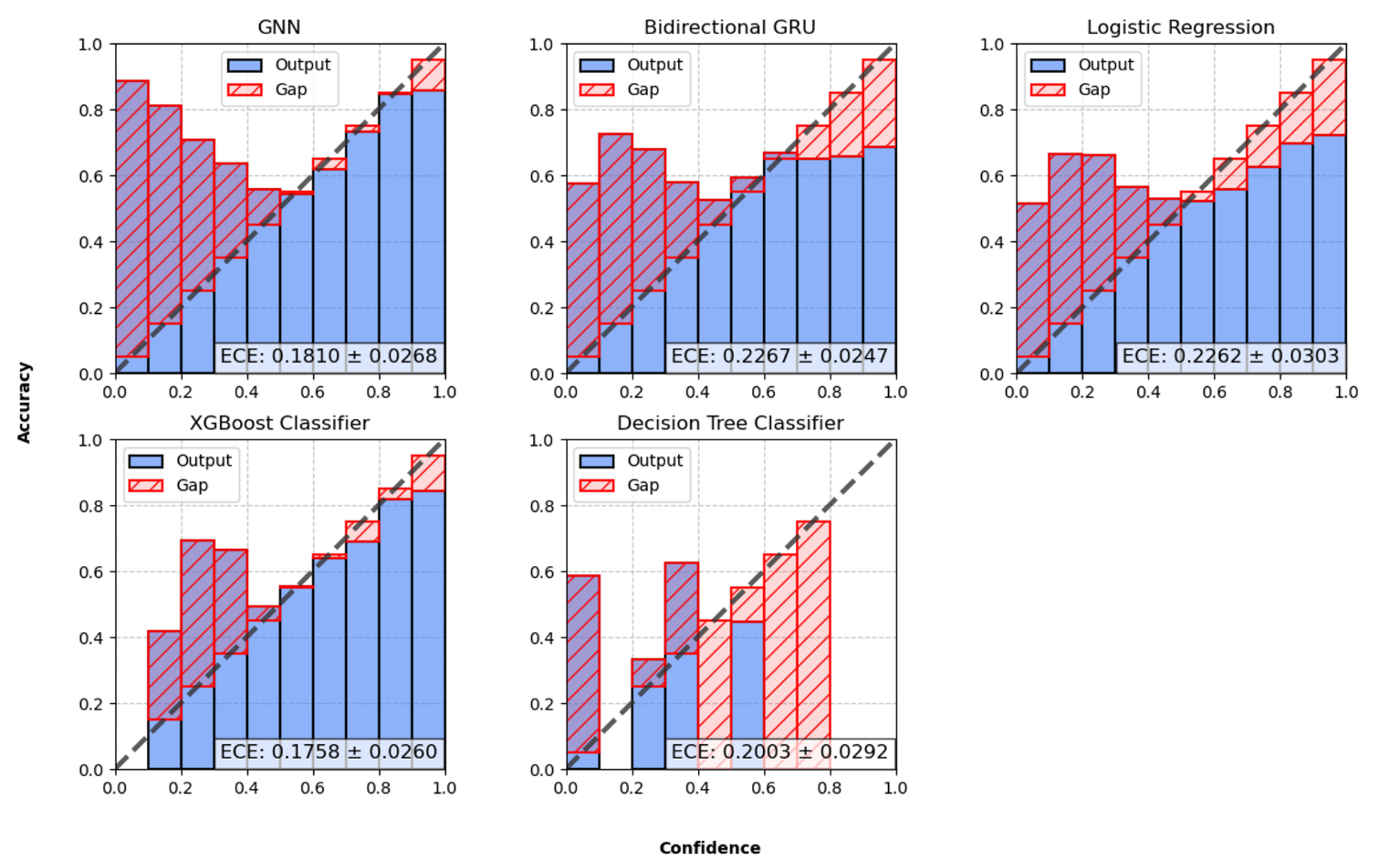
**

Figure A1**.** Reliability diagram and expected calibration error (ECE) for GNN, RNN, conventional machine learning and logistic regression models.

References

1. Naeini MP, Cooper GF, Hauskrecht M. Binary classifier calibration using a Bayesian non-parametric approach. In Proceedings of the 2015 SIAM International Conference on Data Mining 2015; pp. 208-216. Society for Industrial and Applied Mathematics.
2. Guo C, Pleiss G, Sun Y, Weinberger KQ. On calibration of modern neural networks. In International conference on machine learning 2017; pp. 1321-1330. PMLR.

Available from <https://proceedings.mlr.press/v70/guo17a.html>.

1. Wang X, Liu H, Shi C, Yang C. Be confident! towards trustworthy graph neural networks via confidence calibration. Advances in Neural Information Processing Systems. 2021;34:23768-79.
